
JOURNAL INFORMATION
==============================
NLM Title Abbreviation: J Cell Mol Med
Journal ID: J. Cell. Mol. Med
NLM Journal ID: 101083777
Journal ID: jcmm

Journal of Cellular and Molecular Medicine

ISSN: 1582-1838
EISSN: 1582-4934
Publisher: Wiley

ARTICLE INFORMATION
==============================
PMCID: PMC3823418
DOI: 10.1111/j.1582-4934.2012.01430.x
Article version: 1
Subjects: Editorial Board

Issue Information

Print publication date: 2012 Oct
Electronic publication date: 2012 Sep 26
Volume: 16
Issue: 10
First page: i
Last page: ii
Copyright: Copyright © 2012 Foundation for Cellular and Molecular Medicine/Blackwell Publishing Ltd
Copyright year: 2012
License: Re-use of this article is permitted in accordance with the Creative Commons Deed, Attribution 2.5, which does not permit commercial exploitation.
License URL: https://creativecommons.org/licenses/by/2.5/

==============================
### Journal of Cellular and Molecular Medicine

5-year Impact Factor: 5.818

**Editor-in-Chief: L.M. Popescu**
Head, Department of Cellular and Molecular Medicine, Faculty of Medicine
'Carol Davila' University of Medicine and Pharmacy, Bucharest, Romania
Tel: +40 744 535 298; Fax: +40 21 312 4885; E-mail: LMP@jcmm.org

### Editorial Board

**P. Anversa,** Boston, USA
**J.J.M. Bergeron,** Montreal, Canada
**M. Bhatia,** Christchurch, New Zealand
**O. Binah,** Haifa, Israel
**H.E. Blum,** Freiburg, Germany
**T.B. Bolton,** London, UK
**C. Bona,** New York, USA
**M.D. Bootman,** Cambridge, UK
**N. Bornstein,** Tel Aviv, Israel
**G. Bussolati,** Turin, Italy
**K. Camphausen,** Bethesda, USA
**M. Caraglia,** Naples, Italy
**Y.H. Chen,** Shanghai, China
**S.N. Constantinescu,** Brussels, Belgium
**P. Doevendans,** Utrecht, The Netherlands
**B. Eyden,** Manchester, UK
**M.S. Faussone-Pellegrini,** Florence, Italy
**W. Franke,** Heidelberg, Germany
**L. Frati,** Rome, Italy
**T. Fujimoto,** Nagoya, Japan
**P.M. Glazer,** New Haven, USA
**J. Gooch,** Atlanta, USA
**S.K. Gupta,** New Delhi, India
**H. zur Hausen,** Heidelberg, Germany
**A.M. Hofer,** Boston, USA
**R.E. Horch,** Erlangen, Germany
**O. Hovatta,** Huddinge, Sweden
**S.S. Hu,** Beijing, China
**J.D. Huizinga,** Hamilton, Canada
**M. Ivan,** Indianapolis, USA
**B. Jena,** Detroit, USA
**Y.T. Konttinen,** Helsinki, Finland
**T. Kornberg,** San Francisco, USA
**S. Kostin,** Bad Nauheim, Germany
**R.C. Kukreja,** Richmond, VA, USA
**R. Langer,** Cambridge, USA
**J.W. Lawler,** Boston, USA
**F. Lupu,** Oklahoma City, USA
**J. Meldolesi,** Milan, Italy
**M. Mercola,** San Diego, USA
**V.M. Miller,** Rochester, USA
**K. Morgan,** Boston, USA
**J.F. Morris,** Oxford, UK
**C. Mummery,** Utrecht, The Netherlands
**F. Murad,** Houston, USA
**D.F. Mureşanu,** Cluj-Napoca, Romania
**H. O'Neil,** Canberra, Australia
**K.P. Nephew,** Bloomington, USA
**M. Pesce,** Milan, Italy
**O.H. Petersen,** Liverpool, UK
**N.C. Popescu,** Bethesda, USA
**D. Pozo,** Sevilla, Spain
**M.Z. Ratajczak,** Louisville, USA
**U. Ripamonti,** Johannesburg, South Africa
**J. Rubin,** Bethesda, USA
**A. Samali,** Galway, Ireland
**P.R. Sanberg,** Tampa, USA
**R.C. dos Santos Goldenberg** Rio de Janeiro, Brasil
**M. Simionescu,** Bucharest, Romania
**G.W. Sledge,** Indiana, USA
**R.V. Stan,** Hanover, USA
**G. Steinhoff,** Rostock, Germany
**M. Stürzl,** Erlangen, Germany
**M. Taggart,** Newcastle upon Tyne, UK
**A. Tosaki,** Debrecen, Hungary
**N.A. Turner,** Leeds, UK
**C.A. Vacanti,** Boston, USA
**A. Vaheri,** Helsinki, Finland
**L. Vécsei,** Szeged, Hungary
**V. Velculescu,** Baltimore, USA
**C. Wang,** Beijing, China
**R.A. Wang,** Xi'an, China
**B. Winblad,** Stockholm, Sweden
**C. Zhang,** Newark, USA

### Executive Assistant Editor:

**M. Leabu,** Bucharest, Romania

### Executive Assistant Editor:

Asia Office
**X. Wang,** Shanghai, China

### Assistant Editors:

**H. Amin,** Houston, USA
**K. Cheng,** Los Angeles, CA, USA
**L. Chirieac,** Boston, USA
**Sanda M. Ciontea**, Bucharest, Romania
**V. Cismasiu,** Oxford, UK
**Camelia Iancu-Rubin,** New York, USA
**Elisa-Anamaria Liehn,** Aachen, Germany
**N.I. Moldovan,** Columbus, USA
**M. Oprica,** Stockholm, Sweden
**A.G. Papavassiliou,** Athens, Greece
**R.A. Towner,** Oklahoma City, USA

### Publishing Editors:

**D. Cretoiu,** Bucharest, Romania
**C.G. Manole,** Bucharest, Romania

### Consultant Editor:

**M.E. Hinescu,** Bucharest, Romania

### Editorial Office

*Journal of Cellular and Molecular Medicine*, P.O. Box 35-29, Bucharest 35, Romania.
Tel./Fax: +40 21 312 4885. E-mail: editors@jcmm.org

### Publisher

*Journal of Cellular and Molecular Medicine*, Blackwell Publishing Ltd, 9600 Garsington Road, Oxford, OX4 2DQ UK.
Tel.: +44 1865 776868, Fax: +44 1865 714591.
Production Editor: Jeremy Chua (jcmm@wiley.com)

Copyright © 2012 Foundation for Cellular and Molecular Medicine/Blackwell Publishing Ltd.

INDEXED in: Index Medicus - Medline - Current Contents - Science Citation Index...

Journal of Cellular and Molecular Medicine
(Online) ISSN 1582-4934

### Journal of Cellular and Molecular Medicine
### A Journal of Translational Medicine

The *Journal of Cellular and Molecular Medicine* is an international journal publishing peer-reviewed articles dedicated to original research and concepts in all fields of cellular and molecular medicine. Bridging physiology and cellular medicine, and molecular biology and molecular therapeutics, *Journal of Cellular and Molecular Medicine* publishes basic research that furthers our understanding of the cellular and molecular mechanisms of disease and translational studies that convert this knowledge into therapeutic approaches. The journal welcomes Original Articles, Reviews (which may be in Review Series), Short Communications, Editorials and Points of View, as well as Letters to the Editor. From time to time the journal publishes Retro-views of significant ideas and discoveries, and Commentaries.

### Copyright and Photocopying

Journal of Cellular and Molecular Medicine © 2012 Foundation for Cellular and Molecular Medicine/Blackwell Publishing Ltd. All rights reserved. No part of this publication may be reproduced, stored or transmitted in any form or by any means without the prior permission in writing from the copyright holder. Authorization to photocopy items for internal and personal use is granted by the copyright holder for libraries and other users registered with their local Reproduction Rights Organisation (RRO), e.g. Copyright Clearance Center (CCC), 222 Rosewood Drive, Danvers, MA 01923, USA (www.copyright.com), provided the appropriate fee is paid directly to the RRO. This consent does not extend to other kinds of copying such as copying for general distribution, for advertising and promotional purposes, for creating new collective works or for resale. Special requests should be addressed to: permissionsuk@wiley.com

### Disclaimer

The Publisher, Foundation for Cellular and Molecular Medicine and Editors cannot be held responsible for errors or any consequences arising from the use of information contained in this journal; the views and opinions expressed do not necessarily reflect those of the Publisher, Foundation for Cellular and Molecular Medicine and Editors, neither does the publication of advertisements constitute any endorsement by the Publisher, Foundation for Cellular and Molecular Medicine and Editors of the products advertised.

### Wiley's Corporate Citizenship initiative statement

Wiley's Corporate Citizenship initiative seeks to address the environmental, social, economic, and ethical challenges faced in our business and which are important to our diverse stakeholder groups. Since launching the initiative, we have focused on sharing our content with those in need, enhancing community philanthropy, reducing our carbon impact, creating global guidelines and best practices for paper use, establishing a vendor code of ethics, and engaging our colleagues and other stakeholders in our efforts.

Follow our progress at www.wiley.com/go/citizenship

Journal of Cellular and Molecular Medicine is published in 12 issues per year in an online-only format available at Wiley Online Library. Visit wileyonlinelibrary.com to search the articles and register for table of contents e-mail alerts. Institutional subscription prices for 2012 are:
Online only: US$429 (US), US$456 (Rest of World), €270 (Europe), £212 (UK). Prices are exclusive of tax. Asia-Pacific GST, Canadian GST and European VAT will be applied at the appropriate rates. For more information on current tax rates, please go to www.wileyonlinelibrary.com/tax-vat. The price includes online access to the current and all online back files to January 1st 2008, where available. For other pricing options, including access information and terms and conditions, please visit www.wileyonlinelibrary.com/access.

Access to this journal is available free online within institutions in the developing world through the HINARI initiatives with the WHO. For information, visit www.healthinternetwork.org

This journal is indexed by Science Citation Index Expanded, Current Contents/Life Sciences, Journal Citation Reports/Science Edition, Biological Abstracts, BIOSIS Previews.

ISSN (1582-4934) (Online)

For submission instructions, subscription and all other information visit: http://onlinelibrary.wiley.com/journal/10.1111/(ISSN)1582-4934

WILEY-BLACKWELL
